# Microbial diversity in full-scale water supply systems through sequencing technology: a review

**DOI:** 10.1039/d1ra03680g

**Published:** 2021-07-22

**Authors:** Wei Zhou, Weiying Li, Jiping Chen, Yu Zhou, Zhongqing Wei, Longcong Gong

**Affiliations:** College of Environmental Science and Engineering, Tongji University Shanghai 200092 China 123lwyktz@tongji.edu.cn; State Key Laboratory of Pollution Control and Resource Reuse, Tongji University Shanghai 200092 China; Fuzhou Water Affairs Investment Development Co., Ltd. Fuzhou 350000 Fujian China; Fuzhou Water Co., Ltd. Fuzhou 350000 Fujian China

## Abstract

The prevalence of microorganisms in full-scale water supply systems raises concerns about their pathogenicity and threats to public health. Clean tap water is essential for public health safety. The conditions of the water treatment process from the source water to tap water, including source water quality, water treatment processes, the drinking water distribution system (DWDS), and building water supply systems (BWSSs) in buildings, greatly influence the bacterial community in tap water. Given the importance of drinking water biosafety, the study of microbial diversity from source water to tap water is essential. With the development of molecular biology methods and bioinformatics in recent years, sequencing technology has been applied to study bacterial communities in full-scale water supply systems. In this paper, changes in the bacterial community and the influence of each treatment stage on microbial diversity in full-scale water supply systems are classified and analyzed. Microbial traceability analysis and control are discussed, and suggestions for future drinking water biosafety research and its prospects are proposed.

## Abstract 2

Drinking water microbial diversity influence in full-scale water supply systems.

## Graphical abstract

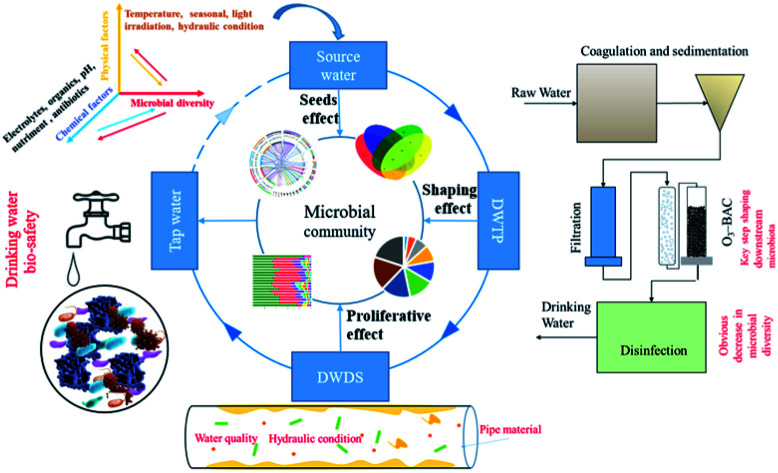

## Introduction

Water security is not only an ecological and environmental issue, but also an economic, social, and political issue that is directly related to national security. Safe drinking water is essential to public health and an integral part of effective policies to protect health.^1^ However, as of 2017, billions of people worldwide still do not have access to safe drinking water and basic health services. According to the World Health Organization (WHO), 80% of human diseases and 50% of child deaths worldwide are related to drinking water quality. With the outbreak of COVID-19, the safety of microorganisms in the environment, especially in water, has become a greater concern.^2–4^ Unlike chemical pollution, microbial pollution is proliferative, secondary, and infectious. The explosive proliferation of microorganisms can result in deterioration of water quality, and the presence of odor or toxins,^5,6^ and induce secondary pollution. Water-mediated pathogenic microorganisms can be transmitted through diet, aerosols, and contact, endangering human health.

Culture-based methods are one of the most widely used traditional analytical approaches to evaluate the microbiological quantity of drinking water. However, due to the overlooked of some bacteria (*e.g.*, viable but non-culturable (VBNC) bacteria), the culture-based method leads to an underestimation of the microbial density and diversity in drinking water.^7^ As a result, nucleic acid-based approaches have been widely applied in recent investigations of drinking water distribution system (DWDS) microbial communities.^8,9^ These culture-independent methods, such as sequencing technology, include internal transcribed spacer (ITS) fingerprints, terminal restriction fragment length polymorphisms (T-RFLP), 16S rRNA gene surveys, and metagenomics surveys,^10^ which could not only detect low concentrations of microorganism (including VBNC), but also obtain microbial diversity information, providing a good technical support for the study of microbial fate.

Given the advantages, sequencing technology, especially high-throughput sequencing (HTS), is widely used to analyze microbial diversity in drinking water to obtain a more comprehensive understanding of bacterial ecology. This review paper summarize the findings of microbial community analysis in full-scale water supply system through sequence technology, especially from the perspective of biological safety of the tap water through distribution system, focusing on (i) the development of sequencing technologies and influence on the study the microorganism in water supply system; (ii) the microbial diversity and environmental impact on full-scale water supply systems using sequencing technology; (iii) evaluation of microbial safety in water source, water treatment process as well as the drinking water distribution system (DWDS); and (iv) proposed biosafety assurance measures for a full-scale water supply system. The goals of this review are to understand application of sequencing technology in the study of drinking water microbial communities, analyze the possible causes of microorganism safety problems in full-scale water supply systems, guide operational practices to obtain safe drinking water, and enhance future research on the drinking water microbiome.

## Development of sequencing technology and its contribution to drinking water investigation

### Development of sequencing technology

**Fig. 1:**
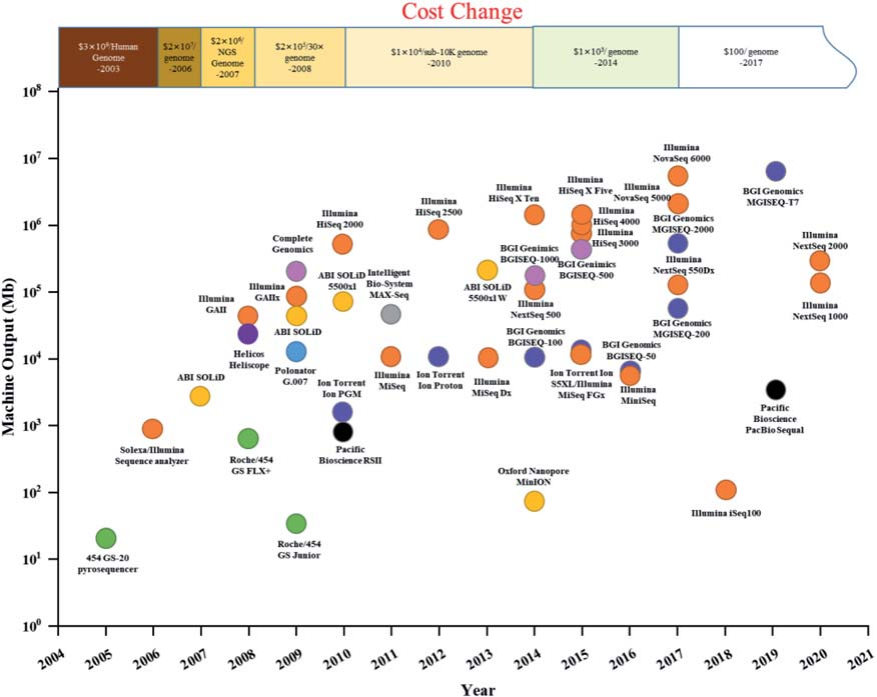
Timeline and comparison of commercial HTS instruments and costs since 2003. (A plot of commercial release dates *versus* machine output per run are shown. For MinION, output from an 18 hour run was used; for MGISEQ-17, output from a one-day run was used. Different dot colours indicate different companies.).

Gene sequencing technology has developed in the last 50 years as a result of the pioneering Sanger and Coulson chain termination method. With the high cost and low throughput of first-generation sequencing technology,^11^ continuous technological development and improvement yielded Roche's 454 technology, Illumina's Solexa technology, and ABI's Solid technology. The comparison of four generation sequencing technologies was list in Table 1. Compared with first-generation Sanger sequencing, they offered high throughput^12^ and fast sequencing, greatly reducing sequencing cost and expanding the scale of genomics research.^13^ The timeline and comparison of commercial HTS instruments and costs since 2003 are shown in Fig. 1. After the introduction of the Genome Sequencer 20 System by 454 Life Sciences in 2005, and the Genome Analyzer II by Illumina/Solexa in 2006, high-throughput sequencing companies were emerging, providing a solid foundation for the development of high-throughput sequencing and price reduction. However, second-generation sequencing technology was still costly, with a short-read length. Since 2008, single-molecule real-time (SMRT, PacBio) sequencing technology and the Heliscope (Helicos Biosciences) genetic analysis system have been developed, known as third-generation sequencing. In NGS method, DNA is broken into short pieces, amplified, and then sequenced. Third generation technologies do not break down or amplify the DNA: they directly sequence a single DNA molecule. Fourth-generation sequencing technology (*e.g.*, Nanopore sequencing technology by Oxford Nanopore Technologies) was invented in 2014.^14^ However, third- and fourth-generation sequencing technologies have relatively lower accuracy and have not been widely used as NGS. Currently, NGS technology is still the predominant sequencing technology in the market. The launch of Illumina's NovaSeq 6000 in 2017 brought the cost of sequencing under $100 per human genome, promoting widespread use of HTS in recent years in medicine, health, and environmental fields (Fig. 1).

**Table 1:** Comparison of four generation sequencing technologies

	First-generation sequencing technology	Next-generation sequencing technology	Third-generation sequencing technology	Fourth-generation sequencing technology
Characteristics	Dideoxy chain termination method	Sequencing by synthesis	Single-molecule sequencing	Nanopore sequencing
Read length	∼1000 bp	50–300 bp	8–12 kb	∼100 kb
Throughput	Low	High	High	High
Instrument time	Long	Short	Short	Short
Relative cost	High	Relatively low	Low	Low
Advantage	Long read length and high accuracy	High throughput, accuracy, speed, and output	Long read length, high throughput, and high speed	Long read length, high throughput, low cost, high speed, simpleness on sample preparation and analysis
Disadvantage	Low throughput and long instrument time	Short read length	Relatively low accuracy	Relatively low accuracy
Represented platforms	ABI	Illumina	Pacbio	Nanopore

### Sequencing technology application in water supply system

**Fig. 2:**
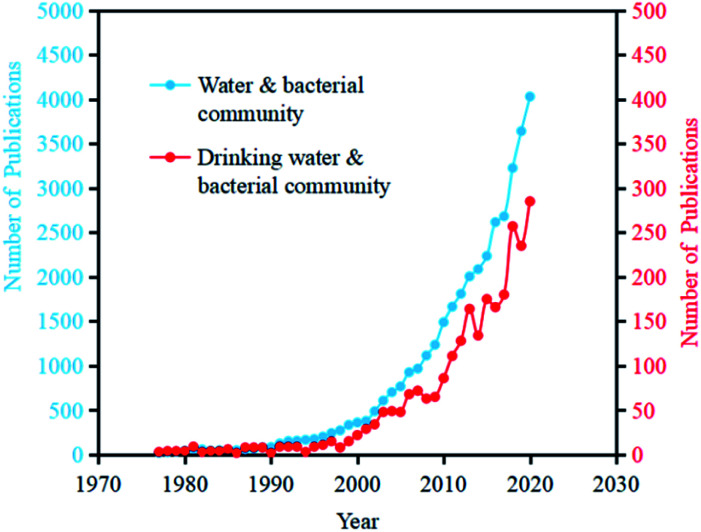
The annual increasing number of publications with the topics of “water & bacterial community” and “drinking water & bacterial community” in Web of Science (as of July 1st, 2021).

With the development of sequencing technology, especially high-throughput technology in recent years, analysis of drinking water microorganisms in relation to human health has been widely conducted.^15^ As the analysis method has gradually shifted from traditional analytical approaches to sequencing analysis methods, the research and focus on the whole process of microorganisms in drinking water has also changed from the original quantity and species to the present diversity, transformation, and function. Searching related papers on the topic of “Water” and “Bacterial community” in Web of Science, the number of publications has reached 39540 (as of July 1, 2021), among which the number of annual increased papers reached more than 1000 since 2007. Searching related papers on the Web of Science with the topic of “Drinking Water” and “Bacterial Community”, the article number has reached 2708 (as of July 1, 2021), among which the growth rate of the increased papers number was largely improved from 2006 (Fig. 2).

Here in this paper, the changes in microbial diversity from source water to tap water were categorized based on reported research which using sequencing technologies, as well as the health risks associated with these changes.

## Impact on microbial diversity in full-scale water supply system

### Water source effects

#### Biological effects

Although the microbial community changes during water treatment, especially in biological and disinfection processes, most microorganisms in drinking water are introduced from those in source water. The diversity of microorganisms in the source water directly affects the species of microorganisms in the drinking water.^16^ Different water sources have different microbial community compositions, resulting in different bacterial communities in the final tap water.^17–21^ Microbial communities are sensitive to changes in their environment and reflect the structure and function of aquatic ecosystems. However, due to the influence of upstream water input and water environment, changes in the water source microbial community are complicated.^22^ The dominant microbial composition may be similar in different water sources, but the abundances may vary.^17^ Delphine,^18^ Pearce^23^ and Henne^24^ found the same microbial compositions, *Actinobacteria*, *Bacteroidetes*, and *Beta-proteobacteria*, in different source waters, in proportions of 40.9%, 22.7%, and 18.2% (Sep Reservoir and Pavin Lake); 19%, 25%, and 26% (Sombre Lake); 16%, 25%, and 20% (two reservoirs in the south of Braunschweig), respectively. The results also showed that the predominant bacterial phyla were *Actinobacteria*, *Proteobacteria*, and *Bacteroidetes*, in reservoirs in Shanghai^22^ and Hong Kong,^25^ with proportions of 46%, 36.6%, and 16.1%; 24.55%, 45.72%, and 14.56%, respectively. Gomez-Alvarez *et al.*^21^ investigated bacterial composition in a metropolitan DWDS using groundwater (GW) and surface water (SW); the results showed that the bacterial diversity of tap water from SW and GW service areas was different, indicating that different source water quality parameters and treatment processes can result in different microbial diversity in the final tap water. As the biological diversity of drinking water sources directly affects the microbial diversity in drinking water, many studies have focused on microbial diversity in drinking water sources and its environmental impacts.

#### Chemical effects

Environmental factors such as temperature,^25–27^ pH,^28,29^ electrolyte type,^19,30,31^ salinity,^32^ dissolved particles,^26^ dissolved oxygen (DO),^33,34^ C/N ratio,^19^ total nitrogen (TN),^25^ total phosphorus (TP),^22^ and organic matter^35,36^ have been investigated, and verified to influence the composition of the microbial community in drinking water. Zhang *et al.*^30^ investigated the bacterial communities during the outbreak and decline of an algal bloom in a drinking water reservoir. The results indicated that the bacterial communities were significantly correlated with conductivity, ammonia nitrogen, water temperature, and Fe. Kaevska *et al.*^26^ found that *actinobacteria* negatively correlated with phosphorus, sulfate, dissolved particles, and chloride levels. *Proteobacteria* positively correlated with sulfate, dissolved particles, chloride, dissolved oxygen, and nitrite levels. Jiang *et al.*^22^ found that the relative abundance of predominant bacteria was affected by environmental factors in source water, and the changes in chemical oxygen demand (COD), TN, and TP in source water were related to microbial diversity. Seasonality also affects the microbial diversity of the source water. Wei *et al.*^25^ found that in a drinking water source in Hong Kong, the microbial community composition and distribution exhibit obvious differences in the dry season and the rainy season, suggesting that seasonal change, as a comprehensive influencing factor, may have a great impact on the microbial diversity of drinking water sources.

#### Organics effects

As early as 1996, Pierre *et al.*^37^ reported the threat of dissolved organic matter (DOM) in water to bacterial regeneration and water treatment. DOM is a mixture of common compounds in drinking water that can affect the optimization and efficiency of water treatment unit operations, including coagulation, sedimentation, and membrane treatment, and serve as the main precursor of disinfection byproducts (DBPs).^38^ Nescerecka *et al.*^35^ found that bacterial proliferation in chlorinated SW samples was restricted mainly by phosphorus and organic carbon; in chlorinated GW samples, carbon was the limiting factor. Apart from some nutrients or DBPs precursors of organic matter, some pharmaceutical and personal care products (PPCPs) which had been widely detected in aquatic environment,^39^ influence the proliferation of bacteria, such as antibiotics,^40,41^ and environmental endocrine disruptors (EEDs).^42,43^ Antibiotics that can screen, enrich, and induce antibiotic-resistant bacteria (ARB) and antibiotic-resistant genes (ARGs) largely affect the stability of microbial diversity, which is a major concern. Deng *et al.*^44^ investigated the antibiotic distribution and microbial diversity in water sources; the results showed that areas polluted with high levels of antibiotics had rich and highly diverse bacterial communities. Ofloxacin posed the main risk to aquatic organisms; the antibiotics in 11.5% of the samples posed resistance selection risks. In recent years, with antibiotics in source water, the investigation of ARGs in full-scale water supply systems has increased, as they may affect the disinfection process in drinking water treatment plants (DWTPs), and the microbial diversity in DWDSs and in tap water. Guo *et al.*^20^ investigated *sul I*, *sul II*, *tet(C)*, *tet(G)*, *tet(X)*, *tet(A)*, *tet(B)*, *tet(O)*, *tet(M)*, *tet(W)*, and 16S rRNA genes in seven DWTPs in the Yangtze River Delta in China. All the investigated ARGs were detected in the source waters of the seven DWTPs; *sul I*, *sul II*, *tet(C)*, and *tet(G)* were the four most abundant ARGs. The total concentration of the sulfonamide or tetracycline resistance gene class was greater than 10^5^ copies per mL. Additionally, Wu *et al.*^36^ studied the influence of disopyramide on bacterial diversity in water; the results showed that the community density and diversity decreased significantly after the addition of disopyramide. In addition, the microbial communities in drinking water sources are affected by antibiotics in water sources.

#### Unconventional water sources effects

In addition to SW and GW, in some areas of water shortage, rainwater,^45,46^ and desalination water^47^ are used as drinking water sources. For rainwater, researchers have used sequencing technology to study the microbial diversity of the water from these sources; the sequencing analysis indicated the presence of one or more fecal indicators, and potential bacterial and protozoan pathogens were detected in the roof-harvested rainwater (RHRW), suggesting that RHRW may not be suitable for drinking. Thus, improving the rainwater biosecurity was proposed through regularly cleaning roofs and gouges, pruning overhanging branches, and reducing the contamination of rainwater tanks by animal waste. For desalination water, the survival microbial pathogens are markedly reduced, especially when comminating with a high level of sunlight radiation. However, some pathogens, such as *Vibrio cholerae*, could still survive. Although most systems could remove the vast majority of microbial pathogens, in some circumstances, there is a significant potential for some pathogens transfer,^10^ thus creates biosafety stress for subsequent processes. Hence, disinfection was recommended whenever possible in these water sources treatments.

**Fig. 3:**
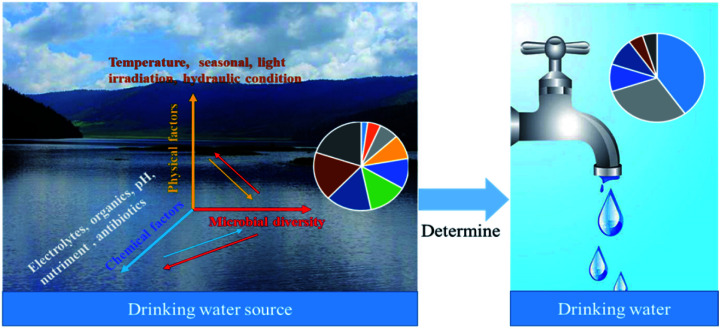
Influencing factors of microbial diversity in drinking water sources and their effects on drinking water biosafety.

The microbial diversity of water sources detected by sequencing technology were categorized in Table 2. Microbial diversity in drinking water sources is influenced by environmental factors, including chemical factors (electrolytes, organics, pH, nutrients, antibiotics) and physical factors (temperature, seasonality, light irradiation). The microbial diversity in the source water also affects the chemical and physical characteristics of the water. In conventional treatment, the source water microbial community is important because it is the source, and the tap water microbial community is the sink (Fig. 3). In studying the dynamic changes of microorganisms in water sources, physical, chemical, and biological properties must be considered together for systematic analysis to evaluate the microorganism diversity more comprehensively.

**Table 2:** The microbial diversity of water sources analyzed by sequencing technology

Factors	Impacts on drinking water microbiome	Ref.
Biological effects	✓ Different water sources have different microbial community compositions, resulting in different bacterial communities in the final tap water	16–18 and 21–25
	✓ The dominant microbial composition may be similar in different water sources, but the abundances may vary	
Chemical and physical effects	✓ Temperature, seasonality seasonal, pH, electrolyte type, salinity, dissolved particles, dissolved oxygen (DO), C/N ratio, total nitrogen (TN), total phosphorus (TP), and COD have been verified to influence the composition of the microbial community in drinking water	19, 22 and 25–36
	✓ *Actinobacteria* negatively correlated with phosphorus, sulfate, dissolved particles, and chloride levels. *Proteobacteria* positively correlated with sulfate, dissolved particles, chloride, dissolved oxygen, and nitrite levels	
	✓ The bacterial diversity was positively correlated with COD_Mn_, turbidity, and pH	
	✓ The bacterial diversity in water source was higher in wet season than in dry season	
Organics effects	✓ Organic nutrients (such as assimilable organic carbon) or DBPs precursors of organic matter have positive effect on the bacteria proliferation	37 and 39–43
	✓ Some pharmaceutical and personal care products (such as antibiotics, and environmental endocrine disruptor) have negative effect on the bacterial diversity, however, they may pose a great threat to drinking water safety	
Unconventional water sources effects	✓ The presence of one or more fecal indicators, and potential bacterial and protozoan pathogens were detected in rainwater, giving suggestion to that it may not be suitable for drinking	17–21 and 45–47
	✓ Disinfection was recommended whenever possible	

### Drinking water treatment processes effects

Drinking water treatment is the key to preventing waterborne diseases and their spread. There is a potential relationship between bacterial community composition and the emergence of opportunistic pathogens;^48^ problems encountered in drinking water treatment plants or water distribution may lead to the proliferation of conditioned pathogenic bacteria (*Mycobacterium*, *Pseudomonas aeruginosa*, *Legionella pneumophila*, *etc.*).^49–51^ Currently, conventional water treatment processes (coagulation–flocculation, sedimentation, filtration, and disinfection) are widely used to purify drinking water in China.^52^ In recent years, with the deterioration of source water quality, especially from an increase in organic matter, advanced treatment technologies such as ozone–biological activated carbon (O_3_–BAC) and membrane treatment have been applied. Understanding changes in the microbial community during treatment is vital for the management of DWTSs. Usually, O_3_–BAC and disinfection processes are regarded as the primary units influencing the microbial density and diversity; other units also have some influence.^53^ A great change in the proportions of *Actinobacteria*, *Proteobacteria*, and *Firmicutes* during the treatment process was detected by Hou *et al.*, the proportion of *Actinobacteria* decreased sharply, and the proportions of *Proteobacteria* and *Firmicutes* increased and predominated in treated water.^54^ During drinking water treatment processes, the microbial activity and bacterial diversity showed obvious spatial differences; the bacterial community changed significantly after chlorination disinfection, indicating that the disinfection process affected the bacterial community. In addition, the bacterial community structure of the finished water was like that of the biofilm on the GAC, indicating that the application of biological treatment technology can significantly change the microbial community composition inherited from the source.

#### Coagulation and sedimentation

Coagulation and sedimentation are the most common processes in water treatment systems to remove microorganisms, such as protozoa (*e.g.*, giardia and crypto) and prokaryotes (*e.g.*, cyanobacteria and bacteria). Here, we'll focus on the effects of the treatment on bacteria. With double electric layer compression, adsorption electric neutralization, adsorption bridging, and sediment trapping, the particulate matter and colloids, and the bacteria adhered to them, are removed from the source water. These processes are generally reported to have no obvious effect on the microbial community structure.^55,56^ However, by monitoring the microbial density and diversity from the influent and effluent of each unit in the water treatment process, Hou *et al.*^54^ found that each unit in the DWTP had an influence on microbial diversity. The removal of microorganisms from water by coagulation and clarification mostly refers to microorganisms that are easily adsorbed on suspended particles and colloids. Strengthening coagulation can greatly reduce the pressure of follow-up disinfection, reducing the cost of follow-up treatment, and reducing the generation of DBPs. Thus, the coagulation and sedimentation process must be considered for microbial safety assurance.

#### Filtration

Filtration (*e.g.*, sand filtration, microfiltration, GAC filtration, and BAC filtration) usually occurs after coagulation and sedimentation; its main function is to intercept the macromolecular solid particles and colloids in water. Filtration is used to remove the suspended matter that has not been removed by coagulation and sedimentation. With good adsorption and interception capability, the filtration process can significantly reduce suspended substances such as bacteria and viruses, further affecting the microbial diversity.^57,58^ In addition, various biological processes (*e.g.*, biofilm formation and shedding) can occur in filters, which could further affect the microbial community structure of the effluent. Bai *et al.*^59^ verified that sand filtering produces a biofilm on the sand that can influence the water quality and microbial diversity. Shaw *et al.*^57^ reported that microfiltration (MF) treatment is the most effective way to inhibit biofilm growth in a DWDS, and that a highly efficient post-treatment disinfection regime reduces the rate of post-treatment regrowth compared with conventional treatment. By investigating the metagenomic characterization of three biofilters (rapid sand filter, GAC filter, and slow sand filter) in a full-scale DWTP, Oh *et al.* found that the bacterial communities in biofilters were significantly different from those in source water and effluent; *Bradyrhizobiaceae* were abundant in GAC, whereas *Nitrospira* were enriched in the sand filters. The GAC community was enriched with functions associated with aromatic degradation, many of which were encoded by *Rhizobiales*.^60^ Lee *et al.*^61^ used q-PCR analysis to clarify ammonia oxidizing bacteria (AOB) and ammonia oxidizing archaea (AOA) effects on ammonium oxidation in a pilot scale rapid sand filter system, the results showed that AOA and AOB were similar in abundance and AOB density set the observed ammonium removal rate. The results were consistence with Tatari *et al.*,^62^ and they also put forward that *Nitrospira* should be the predominant NO_2_ oxidizers. And rapid sand filters are microbially dense, with varying degrees of spatial heterogeneity, leading to in different results, even under very similar experimental setup. In GAC sand filter system, *Nitrosomonas* and *Nitrospira* are likely to be involved in nitrification processes, while *Novosphingobium*, *Comamonadaceae* and *Oxalobacteraceae* may be involved in denitrification processes.^63^ Coincidentally, LaPara *et al.*^64^ also found that AOB were prominent in the bacterial communities, and he most prominent population in the profiles was a *Nitrospira* spp., representing 13 to 21% of the community. By determining the composition of the bacterial community after the stable operation of biological activated carbon (BAC) particles, Zhang *et al.*^65^ found that after nine months of operation, a stable bacterial community dominated by bacteria such as *Pseudomonas* sp., *Bacillus* sp., and *Nitrospira* sp. could effectively eliminate or reduce 41 chemicals in water.

#### O_3_–BAC

When the organic matter in the source water cannot be effectively removed using conventional processes such as coagulation, clarification, and filtration, advanced treatment technology, O_3_–BAC, is often used to minimize the precursor of disinfection byproducts.^66^ However, with the removal of organic matters in DWTP, the leakage of bacteria which could be have been seeding of distribution system, becomes an important issue in this unit. Researchers have increasingly studied microbial diversity changes in the O_3_–BAC unit to determine which microbial consortia colonize filters and what metabolic capacity they possess to obtain the organic matter removal mechanism, based on excellent organic matter removal performance. The influent water quality, oxidative pretreatment, empty bed contact time (EBCT), and backwashing frequency can affect the redox environment of the system, influencing the microbial diversity of the effluent.^71,72^ Soonglerdsongpha *et al.*^73^ compared the O_3_–BAC effect on assimilable organic carbon (AOC) removal in three DWTPs in Japan and found that AOC increased after O_3_ treatment, and BAC could remove 53–73% of the AOC from water, which may be attributed to the microbial community differences. The results were consistent with Liao *et al.*,^74^ who also showed that the BAC filtration system effectively removes both dissolved organic carbon (DOC) and AOC.^76^ Researchers also investigated the effects of temperature,^77^ influent water quality,^76^ types of activated carbon, residence time,^59^ filtration depth, and the backwash process of activated carbon^92^ on the microbial community in the effluent during the operation of O_3_–BAC.

#### Disinfection

The disinfection process is the last barrier to ensure the biological safety of drinking water; its influence on microbial diversity is the greatest in the water treatment process, thus determining the microbial communities in the subsequent units. In recent years, however, chloride-resistant bacteria and VBNC have often been detected in finished water after disinfection, which could result in biofilm formation^78–80^ and pipeline corrosion^81,82^ in the subsequent DWDS. Thus, researchers have studied changes in the microbial community during the disinfection process, focusing mainly on improving the efficiency of disinfection and controlling costs. The results showed that disinfection (chlorination, chloramination, and hypochlorination) has a significant impact on microbial diversity,^83,84^ decreasing bacterial diversity and cultivability, transferring the culturable bacteria from predominantly Gram-negative to predominantly Gram-positive.^93^ After disinfection, alpha- and beta-*proteobacteria* were dominant in chlorinated water. *Betaproteobacteria* was more abundant after chloramine disinfection than the other two processes. The studies also revealed that the richness, diversity, and evenness of bacterial communities were greater in winter than in summer.^94^ Chlorination and chloramination are the two main types of disinfection treatments applied to inactivate pathogens in DWTPs; the efficiency differs based on the disinfectant type and dosage.^85^ Williams *et al.*^86^ compared the bacterial diversity of drinking water in the distribution system after chlorination and chloramination, and found that even after disinfection, numerous bacteria still appeared in the finished water. Although the predominant species in the bacterial community were the same, the microbial diversity was different, which may be due to the difference in the inactivation mechanisms.^81^ The presence of resistant bacteria can accelerate biofilm formation in a DWDS. Researchers have proposed a joint disinfection process^81,82,87^ and develop new disinfectants for the removal of resistant bacteria.^88–90^ In addition, ozone, a strong oxidant is widely used for water treatment, the effect was investigated by Kotlarz *et al.*,^95^ and the results showed that with the detachment of biofilm, the cell concentration in water sample for sequential ozone chambers increased, and biofilms downstream of the dead zone contained a significantly higher relative abundance of bacteria of the genera *Mycobacterium* and *Legionella* than the upstream biofilm. Different from other disinfection method, UV, as a physical disinfection method, is a promising green method and have positive effect on disinfection process when combined with other disinfection method such as UV/Cl_2_,^96,97^ UV/SO_3_^2−^,^98,99^ and UV/H_2_O_2_.^97,100^ Ao *et al.*^101^ investigated the impact of UV treatment on microbial control in DWTPs, the results showed that UV treatment showed high efficacy in inactivating chlorine-resistant microorganisms, and can mitigate microbial re-growth to some extent. *Proteobacteria* (relative abundance: 8.02–92.34%) and *Firmicutes* (1.38–86.87%) were the dominant phyla in UV irradiation samples. Other common phyla included *Bacteroidetes* (1.38–15.26%) and *Actinobacteria* (0.16–8.87%).

**Fig. 4:**
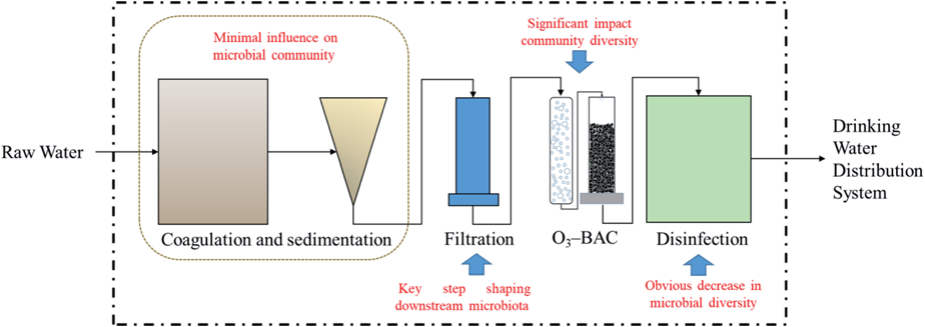
Influence of each unit in DWTP on microbial community.

Drinking water treatment processes effects on its microbiome was counted in Table 3. Generally, drinking water treatment processes have a significant impact on the microbial diversity in tap water. Microbial changes, whether in the traditional processes of coagulation and sedimentation or in subsequent filtration and O_3_–BAC, are adjusted in the disinfection process, resulting in (i) the culturable bacteria transfer from predominantly Gram-negative to predominantly Gram-positive;^93^ (ii) alpha- and betaproteobacteria are dominant in water; (iii) chlorine-resistant bacteria (*e.g.*, VBNC and ARB) may be hidden dangers in subsequent DWDSs. The influence of each unit in the DWTP on the microbial community is shown in Fig. 4. Coagulation and sedimentation have minimal influence on the community; filtration is the key step shaping downstream microbiota. The O_3_–BAC and disinfection processes have the strongest effect in changing the microbial community.

**Table 3:** Drinking water treatment processes effects on its microbiome

Treatment processes	Impacts on drinking water microbiome	Ref.
Coagulation and sedimentation	✓ Early studies suggested that they have minimal influence on the microbial community	54–56, 67 and 68
	✓ With the develop of analytical approach, the results show that they are important for the removal of bacteria in source water	
	✓ The removal of microorganisms from source water by coagulation and clarification mostly refers to microorganisms that are easily adsorbed on suspended particles and colloids	
Filtration	✓ Filtration is the key step shaping downstream microbiota through removing incoming particles and seeding outflow with microorganisms sloughed from filters	57–61, 69 and 70
	✓ The filtration process can significantly reduce suspended substances such as bacteria and viruses, further affecting the microbial diversity	
	✓ Various biological processes can occur in filters	
O_3_–BAC	✓ Ozonation increased taxonomic diversity but decreased functional diversity of the bacterial communities in the BAC filters	71–77
	✓ With the removal of organic matters in DWTP, the leakage of bacteria which could be have been seeding of distribution system, becomes an important issue in this unit	
	✓ The influent water quality, oxidative pretreatment, empty bed contact time, and backwashing frequency can affect the redox environment of the system, influencing the microbial diversity of the effluent	
	✓ O_3_–BAC effect on assimilable organic carbon (AOC) removal in three DWTPs and AOC increased after O_3_ treatment, and BAC could remove most AOC from water, which may be attributed to the microbial community differences	
Disinfection	✓ Different disinfection type and dosage might result in different bacterial populations	78–91
	✓ Generally, after disinfection, alpha- and beta-*proteobacteria* were dominant in chlorinated water. *Betaproteobacteria* was more abundant after chloramine disinfection than the other two processes	
	✓ Although disinfection process could inactivate most bacteria, there are still some chlorine-resistant bacteria existed in finished water, leading to the formation of biofilms in drinking water distribution systems and thus affecting the biosafety of residential water	
	✓ The molecular mechanism of chlorine resistance is attributed to glutathione synthesis	
	✓ Much attentions have been paid on the new approached of disinfection	

### Influence in DWDS

**Fig. 5:**
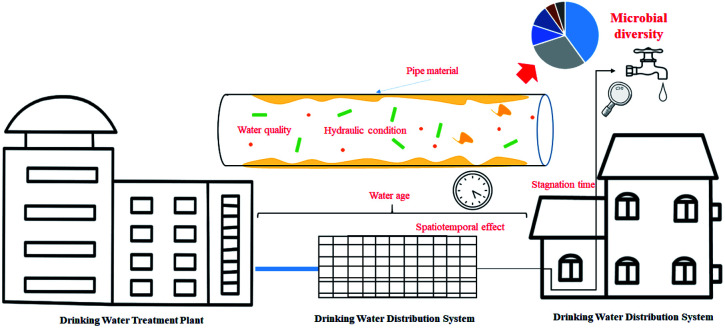
Factors affecting microbiome composition in DWDS.

Mathieu *et al.*^102^ have reviewed the bugs systematically found in drinking water distribution systems all over the world (bacteria, viruses, yeasts, fungi, protozoa, microcrustaceans, rotifers, and oligochaete worms), here, we will analysis and discuss several factors which influence the microbial diversity in DWDS, especially the fate of biofilm on pipes according to the results from HTS. Based on the microbial diversity analysis of finished water and tap water, a diverse core microbiome was shared between the two locations; however, the microbial community was changed in the DWDS,^103^ which was attributed to the shedding of biofilms (the environmental reservoirs for pathogenic microorganisms) from the inner wall of the pipe, posing a potential threat to human health.^104^ Microbial regrowth with spatiotemporal variation is a major concern in distribution, as the physicochemical and nutritional conditions provided by pipe walls are very different from those found during treatment. Recent studies have identified the microbial community and dominant species associated with many factors in the DWDS. These factors include pipe materials, hydraulic conditioning, spatiotemporal effects, and the quality of the treated water (Fig. 5).

#### Pipe materials

To date, the influences of the material and design of DWDS on the biofilm growth on pipe wall have been widely investigated, and these studies have been deepened step by step with the innovation of detection technology. Aggarwal *et al.*^80^ put forward that coupon material (cement, HDPE, and PVC) did not have a significant impact on biomass levels or composition of the biofilm communities in the chloraminated reactors, however, most researchers have given evident on that pipe materials seem to be the most influential factor, followed by spatial and temporal distribution. The pipeline materials influence the density, the formation potential, the formation rate of biofilms, and the microbial diversity. When the biofilm is peeled off from the pipe into the bulk water, it directly affects the microbiome composition in the water. To date, research on the influence of pipe network materials on microbial diversity in drinking water and pipe wall biofilms has focused mainly on cast iron pipes (municipal pipes),^105,110,112^ stainless steel (municipal pipe network into residential area),^116,117^ EPDM and PEX (household plumbing material),^107,113,114^ copper pipe (hotel hot water pipe),^108,109,114,117^ and CP, PVC, and PVCF pipe (household common plastic pipe material).^105,110,112,116,117^ The results showed that the biofilm community structure was different due to the pipe properties, especially for metal pipes. Due to the metal release, the biological diversity in different metal pipes was significantly different, with a greater biological diversity than in plastic materials.^115^ Studies have also shown that the microbiome compositions of biofilms differ in different plastic pipeline materials. The most extensive biofilm was found in HDPE pipes; bacteria adhered to mineral deposits or were immersed in the extracellular polymeric substance (EPS). On the PEX surface, although the bacteria did not form large aggregates, the quantity of bacteria was the greatest. PVC biofilms do not contain mineral deposits, but are composed of single cells rich in *Pseudomonas aeruginosa*, which is harmful to human health.^106^ Roeder *et al.*^107^ found that the biofilm population had greater diversity on growth-supporting materials such as ethylene–propylene–diene monomer (EPDM) than on cross-linked polyethylene. Biofilms are mainly composed of proteobacteria; their composition is influenced by the applied materials. Liu *et al.*^105^ reported that hyphomicrobia and corrosion-associated bacteria were the most dominant bacteria in PVC and cast iron biofilms, indicating that the colonization of bacteria on the material surface was selective. *Mycobacterium* and *Legionella* spp. are common potential pathogenic bacteria in biofilms; however, their proportions were different for PVC and cast-iron pipes. The results also verified that different pipe materials (PVC and cast-iron) have significant effects on the microbial community, especially the bacterial composition. Metal materials such as copper have an antibacterial effect,^109^ which can significantly reduce the microbial diversity downstream. The proportion of bacteria and eukaryotes was reduced by half.^108^ The effects of pipe materials on the drinking water microbiome are presented in Table 4.

**Table 4:** Pipe materials effects on drinking water microbial diversity

Materials	Impacts on biofilms bacterial community	Ref.
PVC and cast ion	✓ Hyphomicrobia was the most dominant bacteria identified in the PVC	105
	✓ Corrosion associated bacteria was the most dominant bacteria identified cast-iron biofilms	
	✓ Bacterial colonization on the material surfaces was selective	
HDPE, PEX and PVC	✓ Coupon material did not have a significant impact on biomass levels or composition of the biofilm communities in the chloraminated reactors	80 and 106
	✓ The biological diversity of different metal pipes was significantly different due to the metal precipitation problem	
	✓ A higher biological diversity was observed in biofilms on metallic material than that on plastic materials	
	✓ The most extensive biofilm was found in pipes of HDPE material	
	✓ The most numerous quantities of bacterial was found in pipes of PEX surface	
EPDM and PEX	✓ The biofilm populations on EPDM were higher than those on PEX	107
Copper	✓ Copper could significantly reduce the microbial diversity downstream	108 and 109
	✓ Effect of copper surface on *Legionella pneumophila* biofilm formation in drinking water	
	✓ Copper could inactivate *Lactobacillus pneumophilus*. In biofilms	
UPVC and copper	✓ Significant differences between bacterial and eukaryotic member in biofilm on UPVC and Cu	110
Epoxying iron, PVC, and cement	✓ Free chlorine was most stable in the presence of PVC while chloramine was most stable in the presence of cement	111 and 112
	✓ The influence of pipe material became apparent at water ages corresponding to low disinfectant residual	
	✓ Each target microbe appeared to display a distinct response to disinfectant type, pipe materials, water age, and their interactions	
EPDM and PEX	✓ Total cell counts and HPC values were highest on EPDM followed by the plastic materials and copper	113
	✓ *P. aeruginosa* and *L. pneumophila* became incorporated into drinking water biofilms on EPDM and PEX	
	✓ Copper biofilms were colonized only by *L. pneumophila* in low culturable numbers	
Copper and PEX	✓ Pipe material seemed to affect mycobacteria occurrence, and bacterial communities with MWT in copper but not in PEX pipes	114
Plastic and stainless steel	✓ The microbiome of biofilms formed on stainless steel and plastics was quite different	115
	✓ Metallic materials facilitate the formation of higher diversity biofilms	
Copper (CU), chlorinated poly vinyl chloride (CP), polybutylene (PB), polyethylene (PE), stainless steel (SS), steel coated with zinc (ST)	✓ Steel pipes (SS and ST) had the highest biofilm formation potential (BFP) and CU showed the lowest BFP	116
	✓ The BFP of CP in drinking water and mixed water were comparable to those of CU	
	✓ PB and PE showed relatively high BFP	

#### Hydraulic conditions

A previous study^118^ reported no statistical difference in microbial communities in biofilms under different hydraulic conditions; biofilms were considered to be a substrate independent of the external environment. However, in bulk water, species richness and diversity were significantly greater in low hydraulic regimes, suggesting that water hydraulic conditions can influence the fate of biofilms. With further excavating to discover the formation mechanism of the biofilm, it was found that the hydraulic condition is related to the formation and shedding of the biofilm, and the water quality in the DWDS.^119^ Thus, it has a great influence on the microbial community of the biofilm and bulk water. Boxall *et al.*^120^ conducted a large number of studies revealing a tendency for greater species richness and diversity with highly varied flow. A more cohesive biofilm structure may be more resistant to external shear stress and detachment. In addition, the flow rate variation during growth was positively correlated with the number of cells, but negatively correlated with the EPS-to-cell volume ratio and bacterial diversity.^121^ The results were consistent with E. Tsagkari's findings, which showed that turbulence could enhance the growth of drinking water biofilms.^122^ Some studies have focused on water discoloration. It was believed that discoloration is influenced by hydraulic conditions,^121,123^ and related to the biofilm shedding in water, indicating that the hydraulic condition plays an important role in the diversity of microbes in drinking water. Additionally, some researchers also argued that the strength of the biofilm matrix is not dictated by the applied fluid shear but is merely coincidental because the EPS composition and density are dictated by other purposes such as a defense from biocides or as a cache of stored food. Thus, one would not expect the strength to increase with fluid shear.

#### Water age

In studying the effects of time on the microbial diversity of drinking water, we considered short-term effects, such as water age or residence time,^124–126^ and long-term effects, such as seasonal changes.^94,127,128^ The results^112,129^ showed that the residual chlorine and DO decreased with the age of the water; DOM, TOC, total bacterial count, and bacterial diversity increased. From the beginning to the end of the DWDS, the relative abundance of *Rhizobium* decreased, and the relative abundance of most other residues increased in varying degrees. Studies have also reported that a greater water age produces a greater relative presence of *M. avium*, which can increase the risk of human infection.^130^ The results were consistent with those of Masters *et al.*^111^ However, in some studies, the effect of water age was not significant. Hwang *et al.*^131^ studied the water-like microbial community at five locations, indicating that at the sampling site and water age (<21.2 h), most of the time samples contained microbes. The composition had no significant effects.

#### Water stagnation

In contrast to municipal water supply systems, water stagnation is an important water supply system characteristic in buildings. In the urban water supply system, the flow in the urban area rarely stops completely due to the high-water demand. However, in buildings, water flow is often stopped for long periods of time, allowing long incubation times for bacteria, and enhancing the formation of biofilms on the inner walls of pipes.^125,132–135^ Stagnation is still an issue in building design. Studying the effect of stagnation time can effectively guide the end-use of water to reduce the risk of microbial contamination. Green-building design often focuses on water conservation, which essentially prolongs water stagnation and accelerates the deterioration of water quality.^136,137^ Studies have shown that the composition of the bacterial community changes dramatically, and the cell count increases by two orders of magnitude after six days of stagnation.^126^ Moreover, the composition and content of microorganisms in household faucet water change greatly, even if stopped overnight.^138^ Chen *et al.*^139^ studied the effect of water stagnation on microbial pollution in a water purifier; the results showed that the growth of microorganisms in the water purifier was faster than in a DWDS, and the size of the microorganisms decreased with an increase in stagnation time. This suggests that microbial contamination caused by stagnation should be carefully considered in the design and usage guidance of building water supply systems (BWSSs) to ensure healthy drinking water.

#### Spatiotemporal effect

The spatiotemporal effect also changes the drinking water microbial community.^34,109,140,141^ Bautista-de Los Santos *et al.*^142^ observed significant changes in the bacterial community over a diurnal time scale and found that the degree and pattern of diurnal changes in the bacterial community in the DWDS were related to the presence/absence of low-content bacteria, and to changes in the relative abundance of dominant bacteria at each sampling site. Perrin *et al.*^143^ found significant but moderate changes in bacterial community composition on large temporal and spatial scales in a drinking water distribution system in Paris. Potgieter *et al.*^94^ found that α-proteobacteria and β-proteobacteria dominated the microbial community in drinking water after disinfection with different disinfectants. In addition, the richness, diversity, and evenness of the bacterial community were greater in winter than in summer. The spatial dynamics of the bacterial community exhibited distance attenuation. However, a survey on the microbial biogeography of drinking water in the Netherlands showed that the population exchange between the biofilm and the water matrix was limited; different DWDSs had different microbial communities, and the treated water had significant stability in time and space.^34^

In addition, treated drinking water quality, including temperature,^132,144,145^ suspended solids,^35,146^ electrolytes,^28,125,147–149^ disinfectants,^149^ and organic matter^28^ also influence microbial diversity in tap water. Sun *et al.*^28^ reported that pH and COD were positively correlated with the relative abundance of *Proteobacteria* and *Firmicutes*. Ma *et al.*^148^ reported that bacterial richness and diversity were positively related to SO_4_^2−^, Cl^−^, and HCO_3_^−^ in the water supply, and negatively related to pH value. Chemical reactions other than microbial processes play a major role in the release of iron during the transition period of the water supply. Moreover, the role of residual chlorine in water quality cannot be underestimated. The disinfectant changes the bacterial community structure of the pipeline biofilm, and affects the water quality and the remodeling of the corrosion scale, further changing the kinetics of the corrosion process.^149^

## Prospects of microbial diversity analysis in drinking water biosafety

With the development of sequencing technology, in-depth characterization and evaluation has been conducted by researchers on microbial communities in DWDSs and BWSSs. Although microbiological safety assurance technology is relatively good, harmful bacteria such as pathogenic bacteria and ARB may still be detected in drinking water, posing a threat to public health.^150–154^ It has been verified that every stage from the source to the tap has some influence on microbial diversity. Thus, researchers have conducted traceability analyses of microorganisms in tap water or estimated the impact on microbial conditions in drinking water based on existing water quality conditions to determine if emergency treatment methods are necessary. Marshall *et al.*^155^ investigated the genotype similarities and geographic relationships of bacterial communities between humans and drinking water. The results indicated that drinking water may be a source of human *Mycobacterium lentiflavum* infection. Liu *et al.*^156^ used the Bayesian “source tracing” method to determine the proportional contributions of source water, treatment water, and the distribution system in shaping the bacterial community in faucet water based on bacterial community fingerprints. The results showed that the source water had no obvious contribution to the bacterial communities of tap water and water in the distribution system. Loose sediments and biofilms show significant effects on phytoplankton and particle-related bacteria in faucet water, which are position dependent and subject to hydraulic changes. In addition, sequencing technology has been used to assess the safety of rural drinking water systems. The rich genetic footprint of pathogens in water samples from many reports suggests that the bacteria can be transmitted to humans. Thus, the importance of disinfection of raw water must be clearly communicated to rural communities to ensure the safe use of water. Studying the microbial community structure and its influence on drinking water is critical.

With the continuous improvement of sequencing technology in depth, accuracy, and economy, how and why the microbial diversity changes in the whole process of drinking water will be clearer. Future research may focus on the impact of new pollutants, traceability analysis and source control, as well as rapid detection and intelligent feedback.

## Conclusion

This paper summarizes and clarifies the biological sequencing technologies applied in research on drinking water microbial communities, including the source drinking water quality, the treatment process, and the distribution system supply conditions, and indicates that all three steps can affect the tap water microbial community. A significant correlation was observed between the microbial populations in the source water and tap water, and the abundance of bacteria was largely affected by the treatment process and the distribution system condition. Thus, the microbiological safety assurance of drinking water must start from the source. The treatment process must be improved, the pipeline network route material must be carefully selected, and drinking water management should be strengthened from the factory to the client, to block the source (water protection), decrease the concentration (optimization of disinfection during DWTP), and control the flow (reduce growth in the DWDS). These mechanisms that need to be explored require the development of cheaper and more accurate biological sequencing technologies.

## Author contributions

The manuscript was written through contributions of all authors. All authors have given approval to the final version of the manuscript.

## Conflicts of interest

There are no conflicts to declare.

## Supplementary Material
